# Knowledge and use of antibiotics among low-income small-scale farmers of Peru

**DOI:** 10.1016/j.prevetmed.2021.105287

**Published:** 2021-04

**Authors:** Julio A. Benavides, Daniel G. Streicker, Milagros S. Gonzales, Elizabeth Rojas-Paniagua, Carlos Shiva

**Affiliations:** a Departamento de Ecología y Biodiversidad, Facultad de Ciencias de la Vida, Universidad Andrés Bello, Santiago, Chile; b Institute of Biodiversity, Animal Health and Comparative Medicine, College of Medical Veterinary and Life Sciences, University of Glasgow, Graham Kerr Building, Glasgow, Scotland, UK; c Centro de Investigación para la Sustentabilidad, Facultad de Ciencias de la Vida, Universidad Andrés Bello, Santiago, Chile; d Millennium Nucleus for Collaborative Research on Bacterial Resistance MICROB-R, Santiago, Chile; e MRC–University of Glasgow Centre for Virus Research, Glasgow, UK; f Facultad de Medicina Veterinaria y Zootecnia de la Universidad Peruana Cayetano Heredia, Lima, Peru

**Keywords:** Livestock, Latin America, Antimicrobial, Tetracycline, Smallholder, Low and middle income country, Cattle

## Abstract

The extensive use and misuse of antibiotics in the livestock sector is one of the main drivers of the emergence and spread of antimicrobial resistance. Although small-scale farms constitute most of the livestock production in low and middle-income countries, knowledge and use of antibiotics among these populations is sparse. We conducted 201 questionnaires to estimate the use and knowledge of antibiotics by small-scale farmers located in the coastal area of the Lima region of Peru. Our results show that farmers had a small number of livestock (e.g. average of 11 cows, 7 pigs and 19 chickens per farm) and 80 % earned less than minimum wage. More than half of farmers reported at least one episode of respiratory disease, diarrhea, mastitis, skin lesion or post-parturition infection in their animals during the previous year, and 40 % of these episodes were treated with antibiotics. Farmers reported using 14 different antibiotics, most commonly oxytetracycline (31 % of episodes treated with antibiotics), penicillin (21 %), gentamicin (19 %) and trimethoprim-sulfamethazine (18 %). The third-generation cephalosporin ceftiofur was occasionally used to treat mastitis. Most farmers relied on veterinarians to prescribe (95 % of respondents) and administer (59 %) antibiotics. Only half of farmers knew what micro-organisms can be treated with antibiotics and the degree of knowledge of antibiotics (based on a 5-question metric) was positively correlated with respondents’ educational level, monthly income, knowledge of the animal health authority, farm area, number of cows and knowledge of an antiparasitic drug. In contrast, knowledge of antibiotics was not correlated with respondents’ age, gender, main occupation, knowledge of a veterinarian or household size. Potential misuse of antibiotics was reported, including 21 % of framers reporting stopping the treatment when clinical signs disappear and infrequent use of antibiotics to treat parasites or animals not eating. Our study highlights poor knowledge and potential misuse of antibiotics among small-scale farmers in coastal Peru, but high reliance on veterinarians for prescription and administration. Strengthening farmers' relationships with veterinarians and improving the diagnostic capacity of the veterinary sector could result in more judicious antibiotic use on these farms.

## 1 Introduction

Antimicrobial resistance (AMR) is a major threat to public and animal health worldwide (FAO, 2016; WHO, 2014). The extensive use of antibiotics in the livestock sector is one of the main drivers of the increase in AMR in agricultural settings over the last decade (FAO, 2016). For example, more than 70 % of antibiotics of medical importance for humans are used in animals in the United States (FDA, 2013; O’ neill, 2016). However, there are still considerable gaps in our understanding of the emergence and spread of AMR in the livestock sector, particularly in low and middle-income countries (LMICs) where small-scale farms comprise the majority of the agriculture sector (Cuong et al., 2018; FAO, 2016; Founou et al., 2016; Lowder et al., 2016). Despite the widespread and intensive use of antibiotics in LMICs such as Brazil and China (Van Boeckel et al., 2015), knowledge of antibiotics among farmers of these countries remains low (Cuong et al., 2018; Redding et al., 2014a). For example, Peru is one of the countries with the highest projected increase in antibiotic use in livestock (Van Boeckel et al., 2015), but only 22 % of small-scale dairy farmers in the Andean region could define what an antibiotic was (Redding et al., 2014a). This lack of knowledge of antibiotic use impedes effective policies aiming to reduce the misuse of antibiotics and thus limit the spread of AMR (Collineau et al., 2017).

Most studies on antibiotic use among livestock from LMICs have focused on dairy farms (Caudell et al., 2017; González Pereyra et al., 2015; Obaidat et al., 2018; Redding et al., 2014a). Similarly to high income countries (De Briyne et al., 2014), common antibiotics used on small-scale farms of LMICs include tetracycline, penicillin, and sulfonamides (Coyne et al., 2019; Cuong et al., 2018; Redding et al., 2014a). Examples include use of penicillin in pig farms of Vietnam and Thailand for both prevention and treatment (Coyne et al., 2019), use of oxytetracycline to treat infections in dairy cattle of Peru (Redding et al., 2014a) and common use of beta-lactamases and aminoglycosides in Argentina (González Pereyra et al., 2015).There is conflicting evidence on whether the amount of antibiotic used per animal in small-scale farms of LMICs is lower than in large farms (Cuong et al., 2018), but even the misuse of a small amount of antibiotics on small-scale farms could increase the emergence and spread of AMR. For example, misuse of antibiotics that were easily purchased without veterinary prescription was reported in small-scale dairy farms of Jordan, where high levels of antimicrobial resistant *Escherichia coli* were reported (Obaidat et al., 2018). To limit the misuse of antibiotics affecting the spread of AMR, it is crucial to understand the factors influencing the knowledge of antibiotics and practices among small-scale farmers of LMICs.

In Latin America, very few studies have quantified the use and knowledge of antibiotics among farmers (Cuong et al., 2018), with only one study in Peru focusing on small-scale dairy farmers (Redding et al., 2014a) and other studies in Argentina and Brazil focusing on larger dairy farms (González Pereyra et al., 2015; Tomazi and dos Santos, 2020). In Peru, antibiotics can be sold and administrated by veterinarians and technicians, but they can also be sold directly by feed stores to farmers without the need of a written prescription nor an established relationship with a veterinarian (Redding et al., 2013). In 2017, Peru launched a national plan to combat antimicrobial resistance using a multi-sectoral One Health approach involving the National Agrarian Health Service (SENASA) from the Ministry of Agriculture and the Ministry of Health (Gobierno del Perú, 2017). This program includes the surveillance of antibiotic use in the livestock sector and improving awareness of antibiotic use through educational campaigns. However, antibiotic use and spread of AMR in the livestock sector remains poorly understood, with a few studies detecting the presence of multidrug-resistant *Salmonella enterica*, methicillin-resistant *Staphylococcus aureus* and extended-spectrum beta-lactamase-producing (ESBL)-*E. coli* among Peruvian livestock (Arriola et al., 2011; Benavides et al., 2018; Vallejos-Sánchez et al., 2019).

The overarching goal of this study was to describe the use, knowledge, and acquisition of antibiotics by low-income small-scale farmers in the peri-urban areas of the Lima region of Peru, where the fecal carriage of ESBL-*E. coli* was previously detected in cows and pigs (Benavides et al., 2018). The Lima region has an estimated livestock population of 289,679 cows, 44,448 pigs, 295,618 sheep, 88,320 goats, 39,046 alpacas and 437,131 backyard poultry (CENAGRO, 2012). This region includes 78,518 farmers working on 75,773 farms extended over approximately 2 million hectares, with 27,718 farmers owning less than half a hectare (CENAGRO, 2012). Farming is the only source of income for 95 % of these farmers. No prior study has quantified the use and knowledge of antibiotics by small-scale farmers in this area. Specifically, we aimed to identify: i) the antibiotic agents commonly used by farmers in the coastal area and the reasons for their use, ii) the person responsible for prescribing and administrating antibiotics, iii) farmers’ level of knowledge of antibiotics, and iv) whether socio-economic and farm characteristics were associated with farmers’ knowledge of antibiotics.

## 2 Material and methods

### 2.1 Study area and participants

A total of 201 farmers were interviewed between September and October 2016, in the districts of Aucallama, (n = 11), Cañete (n = 31), Chancay (n = 33), Huara (n = 26), Mala (n = 1), Pachacamac (n = 34), Paramonga (n = 26) and Pativilca (n = 12) of the Lima region of Peru. Farms were located in peri-urban and rural areas. Farmers were selected from districts where we previously detected ESBL-*E. coli* on livestock and common vampire bats (*Desmodus rotundus*), which feed on the livestock (Benavides et al., 2018). Thus, farms were selected by exploring areas up to 10 km from five previously recorded bat colonies that spread across around 270 km along the coast. Within those areas, we visually identified farms by driving/walking and spotting animals or farming structures from the road. We then approached all farms where the presence of livestock was detected and invited farmers to participate in the study.

### 2.2 Ethics statement

All participants were read a consent form (including study objectives, risks and benefits for participants, confidentiality and that participation was voluntary) and received clarification if requested before giving their oral consent to participate on the study. Participants also received contact information to request study results. The study was approved by the MVLS College Ethics Committee of the University of Glasgow (Application # 200140112) and the Ethics Committee of the veterinary faculty of Universidad Peruana Cayetano Heredia (Application # 012-09-16).

### 2.3 Questionnaires

Questionnaires included 26 questions covering five different parts: 1) farmer socio-economic characteristics and education level, 2) livestock numbers, 3) specific diseases treated or not treated with antibiotics, 4) relationships with veterinarians and SENASA, 5) knowledge of antibiotics, and 6) acquisition and use of antibiotics. The questionnaire was modified from previously available questionnaires (Redding et al., 2014a,b; Benavides et al., 2017). Details on all questions are given on Supplementary Material File S1. Interviews lasted approximately 1 hour and were performed in Spanish or Quechua by E.P. and S.G. Questionnaires were first validated on a small number of farmers (n = 10) to test the clarity of questions and revised where necessary.

### 2.4 Estimation of treated and untreated diseases

We asked farmers the number of animals on their farm (regardless of the species) that had suffered from one or more episodes of clinical disease over the previous year. To compare our results with a previous study conducted in the Andean part of Peru (Redding et al., 2014a), we specifically inquired about five clinical signs: respiratory disease, diarrhea, mastitis, skin lesions and post-parturient infections. For each of the reported episodes, we recorded whether the animal was treated, the type/name of the product used to treat the animal, and if treatment was considered successful.

### 2.5 Antibiotic knowledge score

We built a 5-point knowledge score (KS) which mainly reflected whether farmers knew which micro-organisms can be treated with antibiotics, ranging from 0 (no knowledge) to 4 (good knowledge). The KS was built by integrating the results of five questions. First, we asked a farmer if he or she knew what an antibiotic was. If the farmer gave a negative answer, he/she received a KS = 0 and no further questions were asked. If the farmer gave a positive answer, four other questions were asked. The other four questions included if antibiotics could be used to treat bacteria (yes, KS = 1), viruses (yes, KS = 0), parasites (yes, KS = 0) or fungi (yes, KS = 0). The sum of all these four questions determined the final KS score.

We also asked whether farmers were familiar with seven active ingredients of specific antibiotics that were locally available including tetracycline, chloramphenicol, penicillin, cloxacillin, streptomycin, trimethoprim and florfenicol. Affirmative answers were confirmed by a follow up question which asked if they could name a common product name for this antibiotic available in the local market. As a comparison, we also asked if farmers knew and could name an antiparasitic drug. Given that this information could reflect previous use and familiarity with brands or types of antibiotics rather than an overall knowledge on the action of an antibiotic, these data were analyzed separately from the KS.

### 2.6 Variables correlated with the knowledge score (KS)

We tested the association of the KS with variables describing farmer demography and knowledge including gender, age, education level, monthly income, main economic activity, knowledge of a veterinarian and knowledge of SENASA, and farm characteristics including area (in hectares) and the total number of cows (or other species). To avoid correlation across explanatory variables, we tested the association between the number of each livestock species and KS in separate multivariable models. We report only the model including the number of cows, given that the number of animals for other livestock species (i.e. pigs, sheep, goats, equines, and chickens) was not correlated with KS. The count nature of our response variable (i.e. KS from 0 to 4) required using a generalized linear model with Poisson errors. The model was built using the *glm* function in R 3.6.1 including all variables (R Development Core Team, 2019). The significance of each variable was tested using a Wald’s test within the full model. The *dispersiontest* function from the *AER* package in R indicated that the model was not over dispersed (p > 0.05).

## 3 Results

### 3.1 Characteristics of farmers interviewed

From the 201 farmers interviewed, 131 (65 %) answered all questions. Ten farmers did not want to participate in the study. Most farmers were women (61 %) and were on average 48 [range: 20−87] years old (Table 1). Most farmers raised cattle and grew vegetables (69 %), 24 % exclusively raised animals (19 %) and a small percentage also had other professions including working in a fixed-income job (2%). Only 31 % of farmers finished their secondary school and 80 % had an income lower than 500 Nuevos Soles per month (equivalent to 154 USD with a 3.25 exchange rate in 2017), which is below the minimum wage in Peru (850 Nuevos Soles/262 USD, https://datosmacro.expansion.com/smi/peru). Most farmers (61 %) reported that 0–20 % of their monthly income came from raising animals, while 28 % and 10 % of famers reported higher proportions of income from raising animals, 20–50 % and 50–100 %, respectively. Forty-five percent of farmers sold their animals or derived products exclusively at their farm, 16 % at the market, 15 % at the market or their farm, while 24 % of farmers only used animals for their own consumption. All farmers reported selling their products to traders/merchants. Most farmers (57 %) were familiar with SENASA or a veterinarian (73 %). Farmers reported interacting with SENASA in the context of community visits for livestock vaccination (77 % of respondents), disease diagnostics (12 %), disease control (3 %) and deworming (3 %). The majority (83 %) of farmers had a small farm (less than 1 ha) and had a small total number of animals with a median of 13 (mean = 42.4, range: 1−2000) animals per farm, including 5 (19.7, 0−2000) chickens, 0 (11.1, 0–952) cows, 2 (7.7, 0−500) pigs, 0 (2.2, 2–200) goats, 0 (1–17) sheep and 0 (0.8, 0−46) equines (horses or donkeys) (Table 1).

**Table 1:** Characteristics of 201 small-scale farmers in 8 districts surrounding Lima (Peru) included in this study.

Farmer characteristics	Value
**Number of farmer's interviewed**	201
Number of farmer's answering all questions	131
Percentage of women	61 % (122/201)
Age (mean; median [range])	48.1; 48 [20−87]

**Monthly income (answers n = 173)**	
Less than 500 Nuevos Soles/154 USD	80 % (139/173)
More than 500 Nuevos Soles/ 154 USD	20 % (34/173)

**Education level (n = 200)**	
Uncompleted Primary school	38 % (76/200)
Finished Primary school	31 % (62/200)
Finished Secondary school	31 % (62/200)

**Occupation (n = 165)**	
Farmer	27 % (44/165)
Farmer and another occupation	73 % (121/165)

**Knowledge of SENASA (n = 201)**	
Yes	57 % (115/201)
No	43 % (86/201)

**Knowledge of a veterinarian (n = 201)**	
Yes	73 % (146/201)
No	27 % (55/201)

**Farm size (n = 196)**	
Less than 1 ha	83 % (163/196)
Between 1–3 hectares	14 % (27/196)
More than 3 ha	3% (6/196)

**Number of animals on the farm (n = 201)**	
Total Number of animals	13; 42.4 [1-2000]
Number of chickens (median,mean [range])	5; 19.7 [0−2000]
Number of cows	0; 11.1 [0−952]
Number of pigs	2; 7.7 [0−500]
Number of goats	0; 2.2 [0−200]
Number of sheep	0; 1 [0−17]
Number of equines	0; 0.8 [0−46]

### 3.2 Disease episodes treated with antibiotics

A total of 166 farmers responded to disease-related questions, with 91 farmers (54 %) reporting at least one episode of the five specific diseases asked (Table 2). In the previous year, a total of 470 episodes of these five specific diseases were reported by farmers, with a median of 0 (mean = 2.3, range = 0–50) disease episodes per farm (distributions are provided on Supplementary Material Fig. S2). Farmers reported 138 episodes of respiratory diseases in their animals (94 % treated with any type of medication), 129 episodes of diarrhea (84 % treated), 107 episodes of mastitis (98 % treated), 37 episodes of skin infections (97 % treated) and 59 episodes of post-parturient infection (100 % treated). From a total number of 439 treated episodes, 177 (40 %) were treated with at least one antibiotic. Other treatments included antiparasitic drugs or local remedies including garlic with lemon, salt, mineral oil and water with herbs. A total of 14 antibiotics were used alone or in combination with another antibiotic (Table 2). The most common antibiotics used included oxytetracycline (31 % of episodes treated with antibiotics), penicillin (18 %), gentamicin (19 %) and trimethoprim-sulfamethazine (18 %). The third-generation cephalosporin ceftiofur was used in 7 episodes of mastitis (Table 2). Farmers reported that the treatment worked in all episodes treated with antibiotic and in almost all (98 %) episodes treated with another product.

**Table 2:** Diseases treated with antibiotics on 166 peri-urban small farms in 8 districts surrounding Lima, Peru in 2016.

	**Respiratory disease**	**Diarrhea**	**Mastitis**	**Post-parturient infection**	**Skin infection**
Percentage of farms affected (n)	33 % (55)	34 % (57)	15 % (25)	17 % (16)	15 % (25)
Number of episodes across all farms	138	129	107	59	37
Percentage of episodes treated with any drug (n)	94 % (130)	84 % (109)	98 % (105)	100 % (59)	97 % (36)
Percentage of episodes treated with antibiotics (n)	35 % (45)	36 % (46)	52 % (56)	51 % (30)	0%
Antibiotic used (number of episodes)	Penicillin (15), Oxytetracycline (12), Ampicillin (6), Penicillin/Streptomycin (Penstrep)(5), Enrofloxacin (4), Gentamicin (3)	Gentamicin (30), Oxytetracycline (8), Sulfamethoxazole (3), Penicillin (2), Clindamycin (1), Tylosin (1), Trimethoprim-sulfadiazine (1)	Trimethoprim-sulfadiazine (30), Ceftiofur (7), Oxytetracycline (5), Penicillin (4), Unknown cephalosporin (4), Penicillin/Streptomycin (3), Oxytetracycline/Neomycin (2), Amoxicillin (1)	Oxytetracycline (17), Oxytetracycline/Amoxicillin (10), Penicillin/Streptomycin (3)	None

### 3.3 Acquisition and administration of antibiotics

**Fig. 1:**
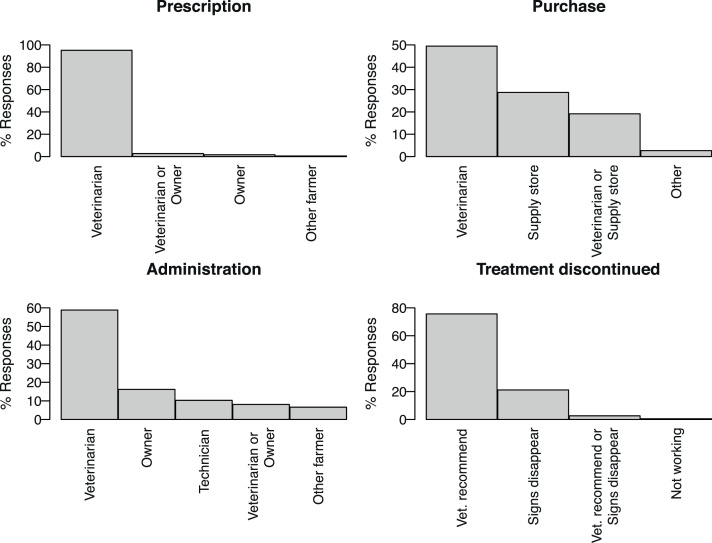
Prescription, acquisition and administration of antibiotics by small-scale farmers of the Lima region, Peru. Each graphic shows the percentage of farmers that responded to a given question including which person prescribed antibiotics (top-left plot), where (or from whom) antibiotics were purchased (top-right), and which person administrated antibiotics (bottom-left). Bottom-right plot shows the main criteria used by each farmer to discontinue the antibiotic treatment.

Almost all farmers (95 %) reported that antibiotics were prescribed exclusively by a veterinarian (Fig. 1). Even if almost all antibiotics were prescribed by veterinarians, farmers purchased the antibiotics from different sources. Indeed, while half of farmers purchased their antibiotics exclusively from veterinarians, 29 % purchased them from a veterinary supply store (also referred as feed store) and 19 % purchased from either a veterinarian or a veterinary supply store. Most farmers (59 %) reported that antibiotics were administrated by a veterinarian, followed by owners (16 %), animal technicians (10 %), veterinarians or owners (8 %) or another farmer (Fig. 1). The majority of farmers (75 %) reported that they followed the duration of treatment recommended by a veterinarian, whereas 21 % reported that they interrupted the treatment when clinical signs disappeared (Fig. 1).

### 3.4 Factors associated with farmer’s knowledge of antibiotics

Farmer’s knowledge score (KS) of antibiotics ranged from 0 (no knowledge of antibiotics) to 4 (farmers knew antibiotics treated bacteria but not viruses, fungi nor parasites). The KS = 0 for 48 % out of 166 farmers, KS = 1 for 22 %, KS = 2 for 11 %, KS = 3 for 12 % and KS = 4 for 7 %. Among farmers not knowing about antibiotics (KS = 0), 15 % still administrated antibiotics to their animals. Several characteristics of farmers and farms were significantly correlated with the KS (Table 3). For example, the KS was significantly higher among farmers that finished primary education (GLM, Odds Ratio (OR) = 2.00 [95 % CI: 1.19–3.43], p < 0.01) and secondary education (OR = 2.74 [1.62–4.73], p < 0.001). Likewise, the KS significantly increased among farmers that earned more than 500 Nuevos Soles per month (OR = 1.69 [1.08–2.64], p = 0.02), farmers owing farms larger than 3 ha (OR = 2.59 [1.03–5.87], p = 0.03), farmers that knew SENASA (OR = 1.77 [1.05–3.06], p = 0.04) and farmers that knew an antiparasitic drug (OR = 2.15 [1.46–3.17], p < 0.0001). The number of cows on a farm was also positively correlated with the KS, but its effect size was small (OR = 1.001 [1.000–1.003], p = 0.04). In contrast, farmer characteristics such as age, gender or main occupation did not correlate with KS (p > 0.05). Likewise, variables including the number of adults in the household, whether the farmer had used antibiotics on his/her animals or whether he/she knew a veterinarian did not correlate with the KS (p > 0.05) (Table 3). Finally, the number of goats, sheep, pigs, equines, or chickens, each tested in a different model including all other variables, did not correlate with the KS (p > 0.05).

**Table 3:** Characteristics of farmers associated with antibiotic knowledge scores in 8 districts surrounding Lima (Peru). Results of a generalized linear model with Poisson error testing the effects of different factors on the knowledge score (0-4) using the glm function in R.

**Variable**	**Estimate**	**Odds Ratio**	**OR** 95 % **CI**	**p-value**
Farmer age	0.01	1.01	1.00;1.03	0.10
Farmer gender	0.37	1.44	0.92;2.27	0.11
**Farmer education (finished primary school)**	**0.70**	**2.00**	**1.19;3.43**	<0.01*
**Farmer education (finished secondary school)**	**1.01**	**2.74**	**1.62;4.73**	<0.001*
Farmer occupation (farming and another)	−0.13	0.88	0.58;1.35	0.55
**Farmer monthly income (more than 500 Nuevos Soles)**	**0.53**	**1.69**	**1.08;2.64**	0.02*
Number of adults on household	0.07	1.07	0.95;1.19	0.27
Farm area (1−3 hectares)	0.26	1.30	0.75;2.14	0.33
**Farm area (more than** 3 ha**)**	**0.95**	**2.59**	**1.03;5.87**	0.03*
**Number of cows on farm**	**0.00**	**1.0010**	**1.000;1.003**	0.02*
**Farmer knows SENASA (yes)**	**0.57**	**1.77**	**1.05;3.06**	0.04*
Farmer knows a veterinarian (yes)	−0.25	0.77	0.48;1.27	0.30
Farmer has used antibiotics on his animals (yes)	0.34	1.41	0.91;2.15	0.11
**Farmer knows an antiparasitic drug (yes)**	**0.77**	**2.15**	**1.46;3.17**	<0.0001*

* Statistical significance considered if p < 0.05.

Regarding specific antibiotics, 26 % (out of 201) of farmers were familiar with the active ingredient and could name a product containing tetracycline, 19 % knew penicillin, 5.5 % trimethoprim, 4.0 % streptomycin, 3.4 % chloramphenicol, 3.0 % cloxacillin and none of the farmers were familiar with florfenicol. In comparison, 23 % of farmers were familiar with an antiparasitic and could name a product containing it. The specific reasons for the use of these antibiotics included unspecified infections (44 % out of 182 reported uses), respiratory problems including pneumonia or coughs (10 %), fever (10 %), mastitis (8.8 %) and diarrhea (8.8 %). Other infrequent reasons for using antibiotics included an animal appearing unwell (3.2 %), treatment of a wound (2.7 %), treatment of parasites (2.2 %), an animal not eating (1%), as preventive treatment (1%) or as a growth factor (0.5 %, one case).

## 4 Discussion

The use and knowledge of antibiotics by small-scale farmers in LMICs is poorly understood, but it is essential in establishing effective measures to reduce the spread of AMR. Our questionnaires showed that farmers in the Lima region of Peru had a small number of livestock (e.g. average of 11 cows, 7 pigs and 19 chickens) and 80 % earned less than minimum wage. More than half of farmers reported an episode of respiratory disease, diarrhea, mastitis, skin lesion or post-parturition infection on their animals during the previous year, and 40 % of these episodes were treated with antibiotics. The most common antibiotics used included tetracycline, penicillin, gentamicin, and trimethoprim -sulfamethazine, although the third-generation cephalosporin ceftiofur was also occasionally used. The relative high cost of ceftiofur compare to other antibiotics used to treat cattle diseases could partially explain its infrequent use. Farmers relied mostly on veterinarians to prescribe, purchase, and administer antibiotics. Knowledge of antibiotics was positively correlated with farmers’ educational level, monthly income, knowledge of the animal health authority (SENASA) and farm features such as area and number of cows.

The most common diseases reported by farmers included diarrhea (34 % of farms affected) and respiratory diseases (33 %). By comparison, dairy farmers in Cajamarca (an Andean region in the northern part of Peru) reported mostly mastitis followed by a similar incidence of diarrhea, respiratory infections, and peri-parturient infections (Redding et al., 2014a). A higher incidence of reported mastitis could result from a higher prevalence of mastitis infections in Cajamarca or from industrial dairy farmers in Cajamarca being more concerned with mastitis than small-scale farmers in Lima. Although we did not quantify disease incidence per animal species or individual, our study shows that farmers are regularly confronted with animals with noticeable clinical signs of disease. In this study, a lower proportion of disease episodes (40 %) were treated with antibiotics compared to the previous study in Cajamarca, where more than 83 % of episodes of similar diseases were treated with antibiotics (Redding et al., 2014a). Lower antibiotic use in this study could be associated with farmers relying less on antibiotics when animals other than cows are sick given their lower economical value, diseases affecting cows in Cajamarca having more severe symptoms that require treatment with antibiotics, or farmers from Cajamarca having a better financial situation related to the dairy industry that allows them to treat disease episodes more often with antibiotics. As in the previous study of Cajamarca, farmers also reported using antiparasitic drugs or local remedies to treat diseases.

Common antibiotics used in this study included oxytetracycline, penicillin, gentamicin and trimethoprim-sulfamethazine. The wide use of oxytetracycline is consistent with previous studies of farmers in other LMICs including Tanzania, Ethiopia and several Asian countries (Caudell et al., 2017; Coyne et al., 2019; Cuong et al., 2018; Gemeda et al., 2020; Redding et al., 2014a). The use of the aminoglycoside gentamicin was reported in large dairy farms of South America such as Argentina and Brazil (González Pereyra et al., 2015; Tomazi and dos Santos, 2020). In Peru, oxytetracycline is widely available in veterinary supply stores under different names and packaging, including fraudulent products (Redding et al., 2013). The wide use of oxytetracycline and penicillin was confirmed by the fact that around 20 % of farmers could remember the market name of these two antibiotics. In addition to being used in the veterinary sector, several of the antibiotics reported by farmers in this study are also important for human medicine including gentamicin, amoxicillin, ampicillin and clindamycin, an antibiotic used to treat bacterial infections caused by Methicillin-resistant *Staphylococcus aureus* (Collignon et al., 2016; WHO, 2019). Therefore, the potential misuse of these antibiotics can also represent a threat of AMR to human health.

The appropriate use of antibiotics by farmers will be determined primarily by whether they are prescribed for the correct agent causing the disease and whether their administration follows adequate guidelines on duration and posology. It is assumed that prudent use of antibiotics by farmers is influenced by several farmer characteristics, economical needs and perceptions, but is also strongly influenced by the advice of veterinarians (Poizat et al., 2017). Our study confirms that small-scale farmers around Lima also relied mostly on veterinarians for acquisition and administration of antibiotics. For example, 95 % of farmer reported that only veterinarians prescribed antibiotics to their animals, while 59 % of farmers reported that antibiotics were exclusively administrated by a veterinarian (compared to 42 % of farmers in Cajamarca (Redding et al., 2014a)). Likewise, 50 % of farmers reported purchasing their antibiotics exclusively from veterinarians, compared to 35 % of farmers in Cajamarca (Redding et al., 2014a). These results suggest that poor small-scale farmers near the capital city of Peru rely heavily on veterinarians to treat their animals with antibiotics, as previously reported for farmers in high-income countries (Friedman et al., 2007; Lathers, 2001). This could simply reflect the greater accessibility of veterinarians to farmers near the capital city. Thus, a potentially effective measure to promote appropriate antibiotic use would be to strengthen the interaction between farmers and veterinarians to ensure the proper diagnosis of the disease agent causing signs observed by farmers. This could help farmers avoid using antibiotics for diseases that do not require them and reduce early stopping of antibiotic treatment before the recommended duration as observed in this study, but should account for their limited resources to acquire the services of a veterinarian (Friedman et al., 2007). Implementing such improved uses of antibiotics may require improving veterinary diagnostic capacity since LMICs often do not have access to adequate laboratory facilities to assist with diagnostics (e.g. access to microbiology laboratories) or testing is not routine (Haider et al., 2017).

Several farmers’ responses suggested potential antibiotic misuse. First, 16 % of farmers reported that they administrated antibiotics to their animals while 29 % of farmers purchased their antibiotics from a veterinary supply store. Although most farmers claim to request a prescription from a veterinarian, purchasing from a veterinary supply store could also mean farmers acquire antibiotics without a prescription from a veterinarian or even professional examination of their animals. In fact, 27 % of farmers responded that they did not know a veterinarian directly, although 95 % of them also indicated that a veterinarian prescribed antibiotics. This contradiction could reflect that some farmers responded what was expected to be the correct answer (i.e. prescription of antibiotics by a veterinarian) instead of their usual practice. Secondly, the correct duration of antibiotic treatment is crucial in avoiding the selection of antibiotic resistance bacteria. There are still many knowledge gaps on the appropriate duration of antibiotic treatment in both human and veterinary medicine, with accumulating evidence showing that shorter durations of antibiotic therapy are sometimes sufficient to treat bacterial infections (Hanretty and Gallagher, 2018; Jessen et al., 2015; Roope et al., 2020). Farmers did not always necessarily follow the recommended duration of treatment, with 20 % of farmers reporting that they interrupted antibiotic when symptoms disappeared. Finally, common antibiotics were mainly used to treat infections, diarrhea and respiratory problems. However, these antibiotics were also infrequently used to treat parasites, animals not eating or animals appearing unwell. This could result in wrongly treating with antibiotics other pathogens that provoke similar symptoms as bacterial infections such as gastroenteritis and respiratory diseases (e.g. coronaviruses (Amer, 2019)).

The frequent veterinary use of the same antibiotic can act as a selective pressure for AMR among domestic animals (Teuber, 2001). Although farmers reported that antibiotics worked in all disease episodes, similar apparent efficacy was observed in almost all episodes treated with other products, raising questions over the reliability of farmers’ assessment regarding disease clearance or reflecting that most of these infections can clear without the need of a specific treatment. Future research should evaluate the effectiveness of antibiotics and whether they are adequately targeting bacterial pathogens. For example, the level of resistance to commonly used antibiotics such as tetracycline, penicillin or gentamicin has not been determined for bacterial pathogens in Peruvian livestock. One previous study detected a high fecal carriage of ESBL- *E. coli* among cows and pigs in this study area (Benavides et al., 2018). The use of third-generation cephalosporins such as ceftiofur could contribute to the selection of ESBL- *E. coli*, but co-selection of ESBL with common antibiotics such as tetracycline, sulfonamide and gentamicin is also possible (Morosini et al., 2006; Tacão et al., 2014). Although reducing antibiotic use is a major measure to reduce AMR, not treating diseases with antibiotics can also have several negative consequences (McEwen et al., 2018), particularly in LMICs with low levels of hygiene and a lack of alternative treatments. Therefore, studies should focus on understanding what factors can increase the appropriate use of antibiotics by low-income farmers.

In this study, farmers’ knowledge of antibiotics was low, with 48 % farmers not knowing what micro-organism can be treated with antibiotics, even if they used them to treat their animals. The drivers influencing the knowledge of antibiotics among small-scale famers in LMICs are poorly known (Redding et al., 2014a), but their identification is essential to increase the adequate use of antibiotics. In this study, we found that higher education levels increased knowledge of antibiotics, similarly to a previous studies on dairy farmers in Cajamarca (Redding et al., 2014a). Knowledge of antibiotics was also positively correlated to the number of cows on the farm and the area of the farm, which could both be associated with a higher economic value of their livestock activity. Knowledge of an antiparasitic also increased the knowledge of antibiotics. A similar pattern of common practices for different diseases was observed in the correlation between livestock vaccination against rabies and vaccination to clostridium among small-scale Peruvian farmers in the Andes (Benavides et al., 2017). Knowledge of SENASA also increased knowledge on antibiotics, reflecting either a better overall knowledge of livestock health by farmers or effective communication about antibiotics by SENASA officials during their visits to vaccinate animals or perform disease diagnostics. Overall, our study highlights the need to increase knowledge of antibiotics among small-scale farmers through effective educational campaigns, ensuring that these campaigns are adapted to their education level and socio-economic status (McEwen and Fedorka‐Cray, 2002). Farmer knowledge about antibiotics has been correlated with appropriate antibiotic use (e.g. reduction of unnecessary use of antibiotics to treat infections that do not require them), and educational campaigns focusing on increasing farmer knowledge can improve the adequate use of antibiotics (Friedman et al., 2007; Raymond et al., 2006). Although such educational campaigns have not been widely developed in the agricultural sector of LMICs and have mainly focused on human medicine (Gozdzielewska et al., 2020; Huttner et al., 2019), a recent study suggests the promising use of digitalized messages to improve antibiotic stewardship among farmers in Bangladesh (Thornber et al., 2019). However, since farmers in our study area rely mostly on veterinarians for antibiotic prescriptions, increasing access to veterinary services could be more effective than campaigns to increase farmer knowledge to improve adequate antibiotic use.

Our study contributes several insights on the knowledge and use of antibiotics among low-income small-scale farmers. However, several limitations of this study could be addressed in future research. First, we only characterized the self-reported number of disease episodes treated with antibiotics, without specific information on incidence per species, antibiotic doses, or duration of treatment. Future studies should quantitatively confirm information provided by farmers to limit reporting bias. For example, longitudinal studies might involve active disease surveillance and monitor antibiotic use data by collecting discarded drug packaging (Redding et al., 2014b). Future research could also disentangle the contribution of each species to antibiotic use within this multi-species agricultural setting. Although we studied specific diseases that can be treated with antibiotics, non-specific signs including lethargy and other diseases such as egg-laying issues or urinary tract diseases that could require antibiotic treatment would not be accounted. However, we expect antibiotic treatment of these other diseases to be less frequent, since chickens and goats do not represent farmers’ main income source from livestock compared to larger animals such as cattle or pigs. Future studies should also standardize a fully validated method to estimate knowledge and explicitly test drivers of prudent antibiotic use that can be compared across studies, as well as how differences in use will affect levels of AMR (Alumran et al., 2012; Collineau et al., 2017; Gemeda et al., 2020). Finally, our study did not investigate knowledge and adherence of antibiotic withdrawal times. Since 76 % of farmers reported selling their animals or animal products, future work could investigate the public health implications of antibiotic residuals and antibiotic resistant bacteria for the consumer on these products.

## Funding

DS and JB were funded by a Sir Henry Dale Fellowship, jointly funded by the Wellcome Trust and Royal Society (Grant 102507/Z/13/Z). CS, JB, NF and DS were also funded by a CONCYTEC‐UK Embassy grant (No. 003‐2016‐FONDECYT). JB was funded by the National Fund for the Scientific and Technological of Chile (FONDECYT-Iniciación, grant number 11181017).

## Declaration of Competing Interest

The authors of this paper have no conflicts of interest to report.

## Figures and Tables

**Figure:**
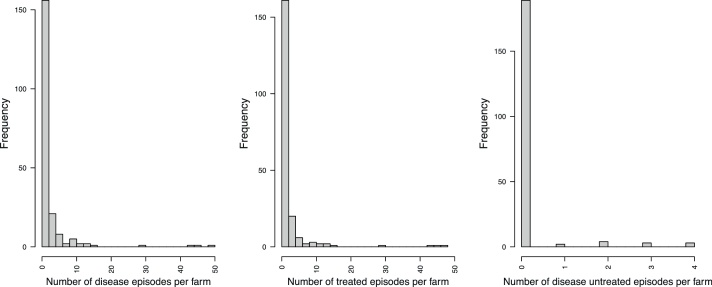


## References

[bib0005] Alumran A., Hou X.Y., Hurst C. (2012). Validity and reliability of instruments designed to measure factors influencing the overuse of antibiotics. J. Infect. Public Health.

[bib0010] Amer H.M. (2019). Bovine-like coronaviruses in domestic and wild ruminants. Anim. Heal. Res. Rev..

[bib0015] Arriola C.S., Güere M.E., Larsen J., Skov R.L., Gilman R.H., Gonzalez A.E., Silbergeld E.K. (2011). Presence of methicillin-resistant Staphylococcus aureus in pigs in Peru. PLoS One.

[bib0020] Benavides J.A., Rojas Paniagua E., Hampson K., Valderrama W., Streicker D.G. (2017). Quantifying the burden of vampire bat rabies in Peruvian livestock. PLoS Negl. Trop. Dis..

[bib0025] Benavides J., Shiva C., Virhuez M., Tello C., Appelgren A., Vendrell J., Solassol J., Godreuil S., Streicker D. (2018). Extended-spectrum beta-lactamases-producing Escherichia coli in common vampire bats and livestock in Peru. Zoonoses Public Health.

[bib0030] Caudell M.A., Quinlan M.B., Subbiah M., Call D.R., Roulette C.J., Roulette J.W., Roth A., Matthews L., Quinlan R.J. (2017). Antimicrobial use and veterinary care among agro-pastoralists in Northern Tanzania. PLoS One.

[bib0035] Collignon P.C., Conly J.M., Andremont A., McEwen S.A., Aidara-Kane A., Griffin P.M., Agerso Y., Dang Ninh T., Donado-Godoy P., Fedorka-Cray P., Fernandez H., Galas M., Irwin R., Karp B., Matar G., McDermott P., Mitema E., Reid-Smith R., Scott H.M., Singh R., Dewaal C.S., Stelling J., Toleman M., Watanabe H., Woo G.J. (2016). Clinical Infectious Diseases.

[bib0040] Collineau L., Belloc C., Stärk K.D.C., Hémonic A., Postma M., Dewulf J., Chauvin C. (2017). Guidance on the selection of appropriate indicators for quantification of antimicrobial usage in humans and animals. Zoonoses Public Health.

[bib0045] Coyne L., Arief R., Benigno C., Giang V.N., Huong L.Q., Jeamsripong S., Kalpravidh W., McGrane J., Padungtod P., Patrick I., Schoonman L., Setyawan E., Sukarno A.H., Srisamran J., Ngoc P.T., Rushton J. (2019). Characterizing antimicrobial use in the livestock sector in three south east asian countries (Indonesia, thailand, and vietnam). Antibiotics.

[bib0050] Cuong N.V., Padungtod P., Thwaites G., Carrique-Mas J.J. (2018). Antimicrobial usage in animal production: a review of the literature with a focus on low-and middle-income countries. Antibiotics.

[bib0055] De Briyne N., Atkinson J., Borriello S.P., Pokludová L. (2014). Antibiotics used most commonly to treat animals in Europe. Vet. Rec..

[bib0060] FAO (2016).

[bib0065] FDA (2013). Food Drug Adm. Dep. Heal. Hum. Serv..

[bib0070] Founou L.L., Founou R.C., Essack S.Y. (2016). Antibiotic resistance in the food chain: a developing country-perspective. Front. Microbiol..

[bib0075] Friedman D.B., Kanwat C.P., Headrick M.L., Patterson N.J., Neely J.C., Smith L.U. (2007). Importance of prudent antibiotic use on dairy farms in South Carolina: a pilot project on farmers’ knowledge, attitudes and practices. Zoonoses Public Health.

[bib0080] Gemeda B.A., Amenu K., Magnusson U., Dohoo I., Hallenberg G.S., Alemayehu G., Desta H., Wieland B. (2020). Antimicrobial use in extensive smallholder livestock farming systems in Ethiopia: knowledge, attitudes, and practices of livestock keepers. Front. Vet. Sci..

[bib0085] Gobierno del Perú (2017). https://antimicrobianos.ins.gob.pe/plan-nacional.

[bib0090] González Pereyra V., Pol M., Pastorino F., Herrero A. (2015). Quantification of antimicrobial usage in dairy cows and preweaned calves in Argentina. Prev. Vet. Med..

[bib0095] Gozdzielewska L., King C., Flowers P., Mellor D., Dunlop P., Price L. (2020). Scoping review of approaches for improving antimicrobial stewardship in livestock farmers and veterinarians. Prev. Vet. Med..

[bib0100] Haider N., Khan S.U., Islam A., Osmani M.G., Rahman M.Z., Epstein J.H., Daszak P., Zeidner N.S. (2017). Efficiency of the clinical veterinary diagnostic practices and drug choices for infectious diseases in livestock in Bangladesh. Transbound. Emerg. Dis..

[bib0105] Hanretty A.M., Gallagher J.C. (2018). Shortened courses of antibiotics for bacterial infections: a systematic review of randomized controlled trials. Pharmacother. J. Hum. Pharmacol. Drug Ther..

[bib0110] Huttner B., Saam M., Moja L., Mah K., Sprenger M., Harbarth S., Magrini N. (2019). How to improve antibiotic awareness campaigns: findings of a WHO global survey. BMJ Glob. Heal..

[bib0115] Jessen L.R., Sørensen T.M., Bjornvad C.R., Nielsen S.S., Guardabassi L. (2015). Effect of antibiotic treatment in canine and feline urinary tract infections: a systematic review. Vet. J..

[bib0120] Lathers C.M. (2001). Role of veterinary medicine in public health: antibiotic use in food animals and humans and the effect on evolution of antibacterial resistance. J. Clin. Pharmacol..

[bib0125] Lowder S.K., Skoet J., Raney T. (2016). The number, size, and distribution of farms, smallholder farms, and family farms worldwide. World Dev..

[bib0130] McEwen S.A., Fedorka‐Cray P.J. (2002). Antimicrobial use and resistance in animals. Clin. Infect. Dis..

[bib0135] McEwen S.A., Angulo F.J., Collignon P.J., Conly J.M. (2018). Unintended consequences associated with national-level restrictions on antimicrobial use in food-producing animals. Lancet Planet. Heal..

[bib0140] Morosini M.I., García-Castillo M., Coque T.M., Valverde A., Novais Â., Loza E., Baquero F., Cantón R. (2006). Antibiotic coresistance in extended-spectrum-β-lactamase-producing Enterobacteriaceae and in vitro activity of tigecycline. Antimicrob. Agents Chemother..

[bib0145] O’ neill J. (2016).

[bib0150] Obaidat M.M., Bani Salman A.E., Davis M.A., Roess A.A. (2018). Major diseases, extensive misuse, and high antimicrobial resistance of Escherichia coli in large- and small-scale dairy cattle farms in Jordan. J. Dairy Sci..

[bib0155] Poizat A., Bonnet-Beaugrand F., Rault A., Fourichon C., Bareille N. (2017). Antibiotic use by farmers to control mastitis as influenced by health advice and dairy farming systems. Prev. Vet. Med..

[bib0160] R Development Core Team (2019).

[bib0165] Raymond M., Wohrle, Call D. (2006). Assessment and promotion of Judicious antibiotic use on dairy farms in Washington State. J. Dairy Sci..

[bib0170] Redding L.E., Barg F.K., Smith G., Galligan D.T., Levy M.Z., Hennessy S. (2013). The role of veterinarians and feed-store vendors in the prescription and use of antibiotics on small dairy farms in rural Peru. J. Dairy Sci..

[bib0175] Redding L.E., Cubas-Delgado F., Sammel M.D., Smith G., Galligan D.T., Levy M.Z., Hennessy S. (2014). The use of antibiotics on small dairy farms in rural Peru. Prev. Vet. Med..

[bib0180] Redding L.E., Cubas-Delgado F., Sammel M.D., Smith G., Galligan D.T., Levy M.Z., Hennessy S. (2014). Comparison of two methods for collecting antibiotic use data on small dairy farms. Prev. Vet. Med..

[bib0185] Roope L.S.J., Buchanan J., Morrell L., Pouwels K.B., Sivyer K., Mowbray F., Abel L., Cross E.L.A., Yardley L., Peto T., Walker A.S., Llewelyn M.J., Wordsworth S. (2020). Why do hospital prescribers continue antibiotics when it is safe to stop? Results of a choice experiment survey. BMC Med..

[bib0190] Tacão M., Moura A., Correia A., Henriques I. (2014). Co-resistance to different classes of antibiotics among ESBL-producers from aquatic systems. Water Res..

[bib0195] Teuber M. (2001). Veterinary use and antibiotic resistance. Curr. Opin. Microbiol..

[bib0200] Thornber K., Huso D., Rahman M.M., Biswas H., Rahman M.H., Brum E., Tyler C.R. (2019). Raising awareness of antimicrobial resistance in rural aquaculture practice in Bangladesh through digital communications: a pilot study. Glob. Health Action.

[bib0205] Tomazi T., dos Santos M.V. (2020). Antimicrobial use for treatment of clinical mastitis in dairy herds from Brazil and its association with herd-level descriptors. Prev. Vet. Med..

[bib0210] Vallejos-Sánchez K., Tataje-Lavanda L., Villanueva-Pérez D., Bendezú J., Montalván Á., Zimic-Peralta M., Fernández-Sánchez M., Fernández-Díaz M. (2019). Whole-Genome Sequencing of a Salmonella enterica subsp. enterica Serovar Infantis Strain Isolated from Broiler Chicken in Peru. Microbiol. Resour. Announc..

[bib0215] Van Boeckel T.P., Brower C., Gilbert M., Grenfell B.T., Levin S.A., Robinson T.P., Teillant A., Laxminarayan R. (2015). Global trends in antimicrobial use in food animals. Proc. Natl. Acad. Sci..

[bib0220] WHO (2019).

[bib0225] WHO, W.H.O (2014). Antimicrobial resistance. Global report on surveillance. World Heal. Organ..

