# Supplementary material for: Knowledge and use of antibiotics among low-income small-scale farmers of Peru
Source: Prev Vet Med. 2021 Apr;189:105287. doi: 10.1016/j.prevetmed.2021.105287 (PMC8636688; doi:10.1016/j.prevetmed.2021.105287)
Supplement: Supplementary file 1 [file mmc1.pdf]

ENTREVISTA A CAMPESINOS ANTIBIOTICO

ID Cuestionario…………………………………… (*Dar un número único, de manera ascendente a cada encuesta*)
Fecha…………………………………………………

Coordenadas GPS del hogar: Latitud……………………. Longitud………………………..

Comunidad…………………………………………………………. **Distrito/Provincia……………………………………………….**

DATOS PERSONALES

Género: Hombre ☐ Mujer Edad………….. **Es jefe de familia:** SI NO ☐

Ingreso mensual (si quiere decir) ☐ 0-200 SOLES ☐ 200- 500 SOLES ☐ 500-1000 SOLES ☐ 1000 +

Porcentaje de su ingreso mensual generado por sus animales 0-20% ☐ 20-50% ☐ 50-100% ☐

Número de adultos que viven en el hogar (mayores de 15 años): Hombres…………… Mujeres…………………

Número de niños que viven en el hogar (menores de 15 años): Hombres………….. Mujeres…………………

Educación del jefe de familia: No terminó primaria ☐ Termino Primaria Termino Secundaria

Estudio en la Universidad o Escuela Técnica, años estudiados:…………………………………………

¿Cuál es la principal ocupación laboral en su vida cotidiana?

Menciona Ganadero ☐ Menciona Agricultor Otra…………………………………

¿De qué tamaño es el predio donde tiene a sus animales?

☐ Predio menos de 1H Predio 1H- 2H- 3H ☐ mas de 3H ☐ Comunal

GANADO

1) **Por favor indíquenos el número de animales que se poseen en el hogar y si convierte sus productos**

Categoría	Número	Leche	Queso	Otro
**Bovinos**				
Bueyes				
Toros				
Novillos				
Vaca adulta				
Vaquilla				
**Ovinos**				
Hembras adultas				
Hembras corderos (aun mamando)				
Machos adultos				
Machos corderos (aun mamando)				
**Caprinos**				
Hembras adultas				

Hembras cabritas (aun mamando)				
Machos adultos (				
Machos cabritos (aun mamando)				
**Chanchos**				
Cerdas de cría (hembra adulta reproductora)				
Cerdos de cría (macho adulto reproductor)				
Cerdos en cebo (ya destetados)				
Chanchitos antes de ser destetados				
**Pollos**				
**Burros**				
Hembras				
Machos				
**Equinos**				
Hembras				
Machos				
**Caninos**				
**Gatos**				
**Cuyes**				
**Otros (conejos, peces, etc) especificar**				

2) ¿En orden de importancia, nos puede indicar cuales son los tres problemas más grandes que considera que tiene para criar a su ganado vacuno?

1er problema………..

2do problema………………………………………………………….3er problema………………………………………………………

3) Dónde y a quién vende generalmente su ganado?

**Animal**	**LUGAR**			**Persona que compro**		
	**Predio**	**Mercado**	**Otro**	**Comerciante**	**Empresa**	**Otro**
Bovinos						
Ovejas						
Cabras						
Cerdos						
**Otros:**						

4) ¿Sabe cuáles fueron las causas de muerte más comunes de sus animales en el último año?

**Animal**	**Número**	**Accidente**	**Enfermedad**	**Otro**	**No sabe**
Bovinos					
Ovejas					
Cabras					
Cerdos					
Pollos					
Cuyes					
Otros:					

SENASA Y VETERINARIO

5) Si a alguien en la comunidad se le muere UNA vaca que estaba enferma, a quien le avisa o a nadie?

☐ *Menciona* No le avisa a nadie *Menciona* Comunicaría al jefe de la comunidad

☐ *Menciona* Avisaría a un veterinario privado /técnico

☐ *Menciona* Avisaría a SENASA *Menciona* Otra………………………………………………

6) Conoce a un técnico veterinario? Si, nombre……………………………………………. ¿Cómo lo contacta?

☐ *Menciona* Guía telefónica ☐ Otro…………………………………………………

☐ *Menciona* Preguntar a otro ganadero ☐ *Menciona* Preguntar a jefe de la comunidad

7) Conoce al SENASA? NO SI, ☐ ¿Última visita a la comunidad?....................................

¿Motivo de la visita?...

ANTIBIÓTICOS

8) ¿Quien realiza los siguientes servicios en su granja?

	**No lo realiza**	**Médico Veterinario**	**Técnico**	**Usted mismo**	**Otro ganadero/vecio**
**Vacunas**					
**Antiparasitarios**					
**Inseminación artificial**					
**Antibióticos**					
**Tratamiento de enfermedades**					

9)

Enfermedad	**# episodios sin tratar (por tipo de animal)**	**# episodios tratados**	**Nombre del producto con el que los trato**	**¿Funciono el tratamiento?**
**Enfermedades Respiratorias**				
**Enfermedades después del parto**				

**Diarrea**				
**Mastitis**				
**Infecciones de piel**				
**Otros**				

10) Sabe lo que es un antibiótico?

SI, ¿qué son?... NO ☐

11) ¿Sabe si los antibióticos se pueden utilizar contra?

Virus SI ☐ NO ☐ Bacterias SI ☐ NO ☐ Parásitos SI ☐ NO ☐ Hongos SI ☐ NO ☐

12) ¿Quien se encarga de recetar los antibióticos que se han de usar en el tratamiento de sus animales?

El médico veterinario ☐

El dueño de los animales ☐

Otros ……….

13) ¿Dónde compra los antibióticos que utiliza en sus animales?

Farmacias y boticas ☐

En agroveterinaria ☐

Directamente al veterinario ☐

A otro ganadero ☐

Promotor/representante

Otros (especificar) ☐ ………………………………………………………..

¿En qué pueblo o ciudad?...

14) ¿Cuándo compra un antibiótico, ¿cuál de estos factores toma en cuenta? (ordene por importancia)

La marca ☐

El lugar donde lo compra ☐

El precio ☐

El envase del producto ☐

La calidad del producto ☐

La recomendación del veterinario o vendedor ☐

☐

La experiencia previa
Otro, ¿Cuál? ……………………………………………….

15) ¿En qué momento suspenderá el tratamiento antibiótico de sus animales?

Cuando finaliza el tiempo de tratamiento recomendado por el fabricante o el veterinario ▢
Cuando el animal se encuentra mejor y no hay más signos clínicos ▢
Cuando aparecen efectos adversos ▢
Cuando se acaba el dinero para el tratamiento
Cuando veo que el tratamiento no funciona
Cuando le baja la producción de leche
Otros (Especificar) ▢ …………………………………………..

16) Conoce alguno de estos productos?

**Producto**	**¿Lo conoce?**	**¿Dónde obtiene?**	**¿Para qué lo utiliza?**	**Nombre del producto**
Oxitetraciclina/Emicina				
Cloramfenicol				
Penicila (Benzatinica,procainica y potásica – Pencivet)				
Cloxacilina (Orbenin)				
Streptomicina				
Trimethoprim/sulfamethoxazole (Sulfatrimetropin - Borgal)				
Florafenicol (Florpro)				
Anti-parasitario				

17) En el último año, ¿Administró antibióticos a sus animales? SI ▢ NO ▢

¿Se acuerda para que enfermedad?..
¿Se acuerda de la marca o nombre del producto? …………………………………………………………………….
